# Continuous 5-fluorouracil infusion plus long acting octreotide in advanced well-differentiated neuroendocrine carcinomas. A phase II trial of the Piemonte Oncology Network

**DOI:** 10.1186/1471-2407-9-388

**Published:** 2009-11-03

**Authors:** Maria P Brizzi, Alfredo Berruti, Anna Ferrero, Enrica Milanesi, Marco Volante, Federico Castiglione, Nadia Birocco, Sebastiano Bombaci, Davide Perroni, Benedetta Ferretti, Oscar Alabiso, Libero Ciuffreda, Oscar Bertetto, Mauro Papotti, Luigi Dogliotti

**Affiliations:** 1 Oncologia Medica, Dipartimento di Scienze Cliniche e Biologiche, Università di Torino, Azienda Ospedaliera San Luigi, Regione Gonzole, 10, 10043 Orbassano (TO), Italy; 2 Anatomia Patologica, Dipartimento di Scienze Cliniche e Biologiche, Università di Torino, Azienda Ospedaliera San Luigi, Regione Gonzole, 10, 10043 Orbassano (TO), Italy; 3 Centro Oncologico Ematologico Subalpino, Azienda Ospedaliera Molinette, Corso Bramante, 88, 10126 Torino, Italy; 4 Oncologia Medica, Ospedale San Lazzaro, Via Pierino Belli, 26, 12051 Alba (CN), Italy; 5 Oncologia Medica, Ospedale di Ivrea, P. Della Credenza, 2, 10015 Ivrea (TO), Italy; 6 Oncologia Medica, Ospedale Civile di Saluzzo, Via Spielberg, 58, 12037 Saluzzo (CN), Italy; 7 Oncologia Medica, Ospedale "B. Eustacchio", Via Del Glorioso, 8, 62027 San Severino Marche (MC), Italy; 8 Oncologia Medica, Azienda Ospedaliera "Maggiore della Carità", Corso Mazzini, 18, 28100 Novara, Italy

## Abstract

**Background:**

Well-differentiated neuroendocrine carcinomas are highly vascularized and may be sensitive to drugs administered on a metronomic schedule that has shown antiangiogenic properties. A phase II study was designed to test the activity of protracted 5-fluorouracil (5FU) infusion plus long-acting release (LAR) octreotide in patients with neuroendocrine carcinoma.

**Methods:**

Twenty-nine patients with metastatic or locally advanced well-differentiated neuroendocrine carcinoma were treated with protracted 5FU intravenous infusion (200 mg/m^2 ^daily) plus LAR octreotide (20 mg monthly). Patients were followed for toxicity, objective response, symptomatic and biochemical response, time to progression and survival.

**Results:**

Assessment by Response Evaluation Criteria in Solid Tumors (RECIST) criteria showed partial response in 7 (24.1%), stable disease in 20 (69.0%), and disease progression in 2 patients. Response did not significantly differ when patients were stratified by primary tumor site and proliferative activity. A biochemical (chromogranin A) response was observed in 12/25 assessable patients (48.0%); symptom relief was obtained in 9/15 symptomatic patients (60.0%). There was non significant decrease in circulating vascular epithelial growth factor (VEGF) over time. Median time to progression was 22.6 months (range, 2.7-68.5); median overall survival was not reached yet. Toxicity was mild and manageable.

**Conclusion:**

Continuous/metronomic 5FU infusion plus LAR octreotide is well tolerated and shows activity in patients with well-differentiated neuroendocrine carcinoma. The potential synergism between metronomic chemotherapy and antiangiogenic drugs provides a rationale for exploring this association in the future.

**Trial registration:**

NCT00953394

## Background

Well-differentiated neuroendocrine carcinomas are a group of rare malignancies generally characterized by low aggressiveness [1-4]. Surgery is the only curative modality. In unresectable disease, somatostatin analogues effectively reduce hormonal hypersecretion, but tumor shrinkage is only rarely seen (0%-6% of patients) [5,6].

Because of their low proliferative activity, conventional chemotherapy for these tumors is not recommended [7-9]. Metronomic chemotherapy, i.e., the frequent administration of cytotoxic drugs at low doses [10], has demonstrated antiangiogenetic properties [11-13]. Since well-differentiated neuroendocrine carcinomas are highly vascular [14,15], there is a rationale for testing metronomic chemotherapy in this clinical setting [16].

Five-fluorouracil (5FU) is frequently employed in the treatment of advanced neuroendocrine carcinoma [17-19]. The combination of protracted 5FU infusion and alfa 2b interferon has been tested in the treatment of well-differentiated neuroendocrine carcinomas of the gastroenteropancreatic tract, and a disease response obtained in more than 40% of patients [20]. Moreover, concomitant administration of somatostatin analogues to enhance the effect of the antiproliferative activity of 5FU has been reported in several *in vitro *and *in vivo *preclinical studies [21].

On these grounds, the Piemonte Region Oncology Network conducted a multicenter phase II trial to assess the activity and safety profile of a combination regimen of protracted 5FU infusion and long-acting release (LAR) octreotide in patients with advanced well-differentiated neuroendocrine carcinomas. The primary study aim was to assess response to treatment; the secondary aims were to evaluate toxicity, biochemical and symptomatic response, time to progression and survival.

## Methods

### Study population

Eligibility criteria included histological diagnosis of well-differentiated neuroendocrine carcinoma according to the World Health Organization (WHO) classification [1], locally advanced or metastatic disease not amenable to surgery with radical intent, at least one measurable target lesion, radiological documentation of progressive disease, somatostatin receptor scintigraphy, Eastern Cooperative Oncology Group (ECOG) performance status grade ≤ 2, life expectancy ≥ 12 weeks, adequate bone marrow reserve (WBC count ≥ 3.5 × 10^9^/L, platelets ≥ 100 × 10^9^/L, hemoglobin ≥ 10 g/dL), adequate hepatic and renal function (hepatic enzymes and bilirubin < 2 × upper limit of normal, serum creatinine < 1.4 mg/dl). Exclusion criteria were non-malignant systemic disease or conditions that precluded patients from receiving the study therapy, second primary malignancies, and previous systemic antineoplastic treatment including somatostatin analogues.

Diagnostic work-up included physical examination, screening chemistry, circulating chromogranin A (CgA), abdominal, chest and pelvis computed tomography (CT), and somatostatin receptor scintigraphy. The study was approved by the local ethic committee of each participating center. Written informed consent was obtained from all patients before starting treatment.

### Histology and immunohistochemical analysis

The histological diagnoses performed at each center were centrally reviewed by two experienced pathologists using the proposed morphological criteria for well-differentiated neuroendocrine carcinoma [1]. In addition, immunohistochemical analysis of the Ki-67 proliferation index (DakoCytomation, Glostrup, Denmark, clone MIB-1, diluted 1/300), type 2 somatostatin receptor expression (BioTrend, Cologne, Germany, code SS-800, diluted 1/3000) and VEGF marker levels (NeoMarkers, Fremont, CA, USA, polyclonal, diluted 1/250) was performed.

### Treatment plan and toxicity assessment

LAR octreotide acetate at a dose of 20 mg was administered intramuscularly every 4 weeks. The first administration was matched to an induction treatment of octreotide (0.1 mg subcutaneous every 8 h) for 14 days; 5FU was given as a protracted continuous infusion without interruption at a daily dose of 200 mg/m^2 ^of body-surface area through an elastomeric pump connected to a central venous access.

5FU treatment was planned to be administered for 6 months, however, prolonged 5FU administration beyond 6 months was permitted. LAR octreotide administration was continued until disease progression.

Toxicity was assessed according to National Cancer Institute Common Toxicity Criteria (NCI CTC) [22].

5FU dose modifications were performed as follows: if WBC count was < 2500/mL and/or platelet count < 100,000/mL, 5FU was delayed for 1 week. If the blood count did not recover, then 5FU was delayed for a further week and the dose reduced by 25%. In the event of severe plantar-palmar erythema with blistering and desquamation, 5FU was interrupted until healing had occurred. In the event of persistent diarrhea and/or grade 2 mucositis, 5FU was discontinued for 1 week. If grade 3/4 diarrhea and mucositis occurred, 5FU was withdrawn until recovery then resumed at a dose reduced by 25%.

### Response evaluation

A CT scan was performed every 12 weeks after initiation of treatment. Disease response was evaluated according to RECIST criteria [23].

Symptomatic patients were asked to keep a diary where they reported frequency and duration of disease-related symptoms. CR was defined as the complete relief of all symptoms and PR as a reduction of at least 50% in both the frequency and intensity of symptoms.

Circulating CgA was measured every 3 months using a commercially available assay (DAKO ELISA, DAKO A/S, Glostrup, Denmark). A decline in CgA levels was considered a biochemical response and defined as either a CR if the tumor marker level returned to normal or as a PR if tumor marker levels decreased by 50% or more. An increase of more than 25% in CgA levels was defined as biochemical progression.

Circulating VEGF levels were retrospectively assessed using a sandwich enzyme immunoassay technique with a monoclonal antibody specific for VEGF (R&D System Europe, Abingdon, UK) in plasma samples collected at baseline and after 3 and 6 months in 11 patients, all recruited at the study coordinating center (San Luigi Hospital, Orbassano, Torino).

### Statistical analysis

The primary end point of the study was the response rate. A Simon two-stage design [24] was used to test the null hypothesis that the true objective response rate was < 10%. The upper limit of first-step drug rejection was 1 response (CR or PR) in the first 10 consecutive patients; the upper limit of second-step rejection was 5 responses in 29 consecutively enrolled patients. Time to progression (TTP) and overall survival (OS) were assessed using the Kaplan-Meier product limit estimate method. The rates of TTP and OS were measured from the date of treatment start to the date of progression and the date of last follow-up or death, respectively. A Cox proportional-hazards regression analysis was used to assess in multivariate analysis the prognostic role of mitosis count and Ki67 expression. Changes in VEGF levels over time were assessed by the Friedman analysis of variance for non parametric matched paired data. P values < 0.05 were considered statistically significant. Statistical analyses were performed using the Statistica/PC software program (Statsoft, Tulsa OK, USA).

## Results

### Patient characteristics

Twenty-nine patients with locally advanced or metastatic disease with radiological documentation of progressive disease were entered into the study at the seven participating centers from 2004 to 2006. The baseline patient characteristics are depicted in Table 1. No patients had received any previous systemic antineoplastic treatment including somatostatin analogues. None had received radionuclide therapy before entry into the study.

**Table 1:** Patient characteristics

Patients	N = 29
**Median age (range) - yrs**	59 (26 -- 80)
**Gender -- no. (%)**	
Male (%)	15 (52.0)
Female (%)	14 (48.0)
**ECOG performance status -- no. (%)**	
0	11 (37.9)
1	17 (58.6)
2	1 (3.5)
**Primary tumor site -- no. (%)**	
Pancreas	13 (44.8)
Functioning	5 (17.2)
Unknown	7 (24.2)
Small bowel	7 (24.2)
Appendix	1 (3.4)
Colon	1 (3.4)
**Tumor Stage -- no. (%)**	
Locally advanced/metastatic	2/27
**Site of metastases -- no. (%)**	
< 2 sites	13 (44.8)
≥ 2 sites	16 (55.2)
**Prior therapies -- no. (%)**	
Surgery	9 (31.0)
Progressive disease	29 (100)
**Baseline symptoms -- no. (%)**	
Diarrhea	6 (20.6)
Flushing	4 (13.8)
Carcinoid syndrome	4 (13.8)
Hypoglycemia	2 (6.9)
Others	10 (34.5)
**Circulating chromogranin A levels (U/L) - no. (%)**	
Normal levels	4 (13.8)
Supranormal levels	25 (86.2)
**Histological tumor characteristics -- no. (%)** **SST2**	
**1+**	2 (9.1)
**2+**	8 (36.4)
**3+**	12 (54.5)
**missing**	7
**Mitosis (per square millimeter)**	
**< 2**	17 (68.0)
**2 -- 20**	7 (28.0)
> **20**	1 (4.0)
**missing**	4
**Ki67**	
**≤ 2%**	11 (42.3)
**3 -- 10%**	13 (50.0)
**> 10%**	2 (7.7)
**missing**	3
**VEGF**	
**0**	3 (15.8)
**1**	4 (21.1)
**2**	7 (36.8)
**3**	5 (26.8)
**missing**	10

SST2 = somatostatin receptor type 2

VEGF = vascular endothelial growth factor

A diagnosis of well-differentiated neuroendocrine carcinoma was confirmed in 28 patients by central histological review. One patient had a poorly differentiated carcinoma and was therefore ineligible. However, according to the intent-to-treat principle, this patient was included in the analyses.

### Treatment and toxicity

5FU treatment was administered for a median duration of 6 months (range, 2-9). Twenty-three patients (79.3%) completed the scheduled 6-month 5FU treatment. Six patients interrupted earlier because of disease progression (2 cases), patient decision (1 case), radionuclide therapy (2 cases), and surgical debulking to control an insulinoma syndrome (1 case). Associated side effects were mild (Table 2). No hematologic grade III-IV events were recorded. Grade II-III mucositis and diarrhea occurred in 3 patients (10.3%). Two patients (6.9%) developed grade III hand-foot syndrome. 5FU administration was delayed for at least 1 week in 8 patients (27.6%) and for 2 weeks in 3 patients (10.3%). The reasons for the delayed course were hematological in 1 patient and non-hematological (mucositis, diarrhea, cutaneous) in the other 10 cases. 5FU infusion was reduced by 75% in 2 patients due to old age and grade III diarrhea and reduced by 50% in 1 patient due to grade III mucositis.

**Table 2:** Treatment-related toxicity.

Higher grade WHO toxicity	N = 29 No. (%)	N = 29 No. (%)	N = 29 No. (%)
	**1**	**2**	**3**
**Anemia**	--	2 (6.9)	**--**
**Thrombocytopenia**	1 (3.4)	**--**	**--**
**Neutropenia**	2 (6.9)	2 (6.9)	**--**
**Asthenia**	9 (31.0)	2 (6.9)	--
**Nausea-vomiting**	7 (24.1)	1 (3.4)	--
**Diarrhea**	16 (55.1)	2 (6.9)	1(3.4)
**Hand-foot syndrome**	4 (13.8)	4 (13.8)	2 (6.9)
**Mucositis**	4 (13.8)	2 (6.9)	1 (3.4)

### Treatment activity

Seven patients attained a PR (24.1%), 20 SD (69.0%), and 2 (6.9%) progressed (Table 3). Disease responses were observed in patients with liver (5 patients) and lymph node metastasis (2 patients).

**Table 3:** Response to treatment

	Clinical response (RECIST) N = 29 No. (%)	Biochemical response (Chromogranin A) N = 25 No. (%)	Symptomatic response N = 15 No. (%)
Progression	2 (6.9)	2 (8.0)	
No change	20 (69.0)	11 (44.0)	6 (40.0)
Partial response	7 (24.1)	8 (32.0)	6 (40.0)
Complete response	**-**	4 (16.0)	3 (20.0)
Overall response	7 (24.1) (95% CI 8.3-39.9)	12 (48.0) (95% CI 28.0-68.0)	9 (60.0)

RECIST = Response Evaluation Criteria in Solid Tumors

Biochemical and symptomatic response was obtained in 48% and 60% of patients, respectively. After medical treatment, surgery was carried out in 3 patients: 1 with a PR and 2 with SD (minimal tumor shrinkage < 20%). One patient was operated for surgical removal of a primary pancreatic tumor and chemoembolization of liver metastases; 1 patient underwent right hemicolectomy plus intraoperative radiofrequency ablation of liver metastases, 1 patient underwent radical excision of a primary pancreatic tumor and liver metastases. Disease response did not change when the patients were stratified by primary disease site, mitotic count and Ki67.

In the 11 patients with circulating VEGF assessed before and after treatment, there was a non significant trend of progressive marker reduction over time (mean VEGF levels 147, 96, and 67 pg/ml at baseline, 3 and 6 months, respectively, P = 0.33). VEGF decreased > 50% from baseline in 5 patients, between 25 and 50% in 2, < 25% in 2, and increased > 25% in 2.

### Predictors of patient outcome

At the last follow-up evaluation, 16 (55.2%) patients showed disease progression and 7 (24.1%) had died; the median duration of follow-up was 51 months.

**Figure 1:**
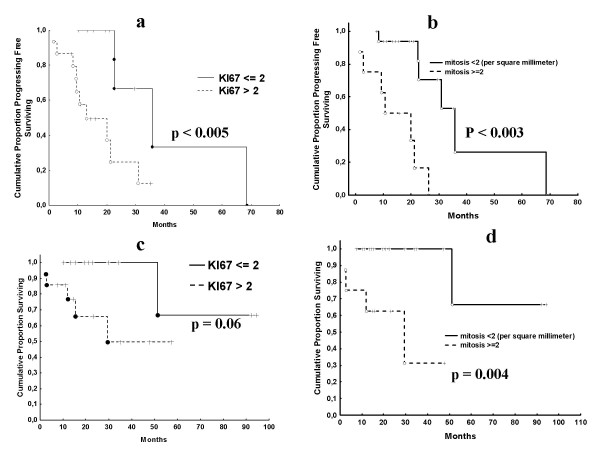
**Time to progression (1a and 1b) and overall survival (1c and 1d) according to Ki67 expression at the cutoff of 2% positive cells and mitosis count**.

The median TTP of all 29 patients was 22.6 months (range, 2.7-68.5), while the median OS was not reached. After 2 years, 48% of patients were free from progression and 82% were alive. In the univariate analysis, Ki67 immunostaining at a cutoff value ≤ 2% and mitotic count at a cutoff value < 2% were predictors of either TTP (P < 0.005 and P < 0.003) or OS (P = 0.06 and P = 0.004) (Figure 1), while age, sex, patient performance status, primary disease site and VEGF expression were not. In the multivariate analysis, Ki67 (continuous variable) but not mitotic count was an independent predictor of either disease recurrence (hazard ratio [HR] 1.03, 95% confidence interval [CI] 1.00-1.06; P = 0.05) or death (HR 1.04, 95% CI 1.00-1.08; P < 0.04). Of the 3 patients who underwent surgery after treatment, only 1 patient progressed after 20.0 months. The remaining 2 patients were alive and free from progression after 29.7 and 35.1 months, respectively.

## Discussion

In this multicenter phase II trial, protracted 5-fluorouracil (5FU) infusion administered in combination with LAR octreotide was an active regimen in the upfront treatment of advanced neuroendocrine carcinomas. This regimen was associated with an overall objective response rate of 24%, a biochemical response rate of 48%, and a symptomatic response rate of 60%. To our knowledge, this is the first trial to test the activity of the combination of octreotide and a chemotherapy drug in somatostatin-naïve patients. Due to the rarity of neuroendocrine tumors, this trial was not randomized and this represents a limitation. Nonetheless, the objective response rate obtained here seems to be higher than that expected with somatostatin alone (0-6%) [4,8,25]. The recently presented results of the first randomized study testing the efficacy of octreotide versus placebo in metastatic well-differentiated neuroendocrine carcinoma showed that octreotide was superior to placebo in prolonging time to progression, despite a comparable response rate [26]. These data further confirm that the antineoplastic activity of somatastatin analogues is not due to tumor shrinkage but rather to a prolonging of disease stabilization. The potentiality of continuous 5FU in inducing tumor shrinkage could improve the efficacy of systemic treatment, a finding that needs to be assessed in a randomized study.

Due to the potential dedifferentiation of neuroendocrine carcinomas as a consequence of progression, it is expected that response to treatment in metastatic sites may differ from that of primary lesions. Our series was homogeneous in this respect, since 27 out of 29 patients had metastatic disease. Tumor shrinkage (partial response or minimal response) obtained by our regimen made surgery for residual disease (with/without liver chemoembolization or liver radiofrequency ablation) feasible in 3 patients. Surgery for residual disease seemed to be efficacious, since 2 patients were disease free at the last follow-up examination after 27 and 35 months, respectively.

In order to verify study population homogeneity, the tumor samples were centrally reviewed by two pathologists. Central review also allowed the pathologists to assess the biological prognostic parameters. Significant outcome predictors were mitotic count and Ki67 proliferation index, both parameters being reciprocally correlated. Only Ki67, however, maintained an independent prognostic role in the multivariate analysis, confirming the importance of assessing this marker in these tumors [27,28]. Ki67 is also an essential parameter in the proposed grading system for foregut neuroendocrine tumors of the stomach, duodenum, and pancreas [29]. The proposed cutoff of 2% to discriminate G1 from G2 carcinomas was associated with better outcome, confirming a recent report [30]. Ki67 is also used as predictive marker of treatment response in clinical decision-making algorithms [7,31]. It has been suggested that neuroendocrine carcinomas with high Ki67 expression may be potentially sensitive to conventional chemotherapy, whereas tumors with lower Ki67 expression may not [31]. This trend was confirmed in our series but failed to attain statistical significance.

It has been suggested that metronomic chemotherapy has antiangiogenetic activity [10]. The activity of metronomic chemotherapy in reducing circulating VEGF levels was observed in a previous study in patients with advanced breast cancer [32]. In the present study, plasma VEGF levels monitored every 3 months in 11 patients showed a stepwise reduction over time that failed to attain statistical significance due to the small sample size. On an individual basis, however, 7 patients were noted to have a decrease in VEGF and only 2 an increase. Taken together, these data evince an antiangiogenic effect of continuous 5FU plus octreotide treatment.

In a recent Italian multicenter study, a combination of oxaliplatin and metronomic capecitabine (an oral 5FU analogue) was administered to 40 patients with malignant neuroendocrine tumors. Interestingly, a disease response was observed in 23% of patients with high-grade and in 30% of those with low-grade neuroendocrine tumors, respectively [33]. The introduction of metronomic capecitabine could account for the high response rate obtained in this well-differentiated subset.

Continuous 5FU infusion in this patient subset was well tolerated as most toxicities observed were mild (grade I or II). As expected, dose-limiting toxicities were non hematolological (mucositis, diarrhea, hand-foot syndrome) and delayed treatment in 40% of patients.

## Conclusion

The combination of 5FU continuous infusion and LAR octreotide is an active regimen in the treatment of metastatic well-differentiated neuroendocrine carcinomas and may have antiangiogenic activity. The potential synergism between metronomic chemotherapy and antiangiogenic drugs provides a rationale for exploring this association in the future. Moreover, capecitabine is an orally available 5FU derivative found to have the same efficacy as continuous 5FU infusion in patients with advanced colon carcinoma. Capecitabine administered on a metronomic schedule instead of 5FU infusion should be tested in patients with advanced neuroendocrine carcinoma, thus obviating the need for an implantable central venous access.

## Competing interests

The authors declare that they have no competing interests.

## Authors' contributions

BMP and BA were critically involved in the study design, statistical analysis, and drafting of the manuscript. DL and BO conceived of the study, participated in designing the study and assisted in drafting the manuscript. VM and PM carried out the immunohistochemical analysis and the central histological review. CF, BN, BS, PD, FB, AO, CL participated in primer selection, attended to patients, and revised the methods section of the manuscript. FA and ME were involved in data collection. All authors have read and approved the final manuscript.

## Pre-publication history

The pre-publication history for this paper can be accessed here:

http://www.biomedcentral.com/1471-2407/9/388/prepub
